# A Retrospective Observational Study on the Comparison of Different Diagnostic Modalities of Post-COVID Mucormycosis

**DOI:** 10.7759/cureus.48925

**Published:** 2023-11-16

**Authors:** Guddi Rani Singh, Shabana Azad, Mamta Kumari, Sweta Kumari, Sanjiv Kumar, Ausaf Ahmed

**Affiliations:** 1 Pathology, Indira Gandhi Institute of Medical Sciences, Patna, IND; 2 Pathology, Homi Bhabha Cancer Hospital, Varanasi, IND; 3 Biochemistry, Indira Gandhi Institute of Medical Sciences, Patna, IND; 4 Microbiology, Indira Gandhi Institute of Medical Sciences, Patna, IND; 5 Surgery, Indira Gandhi Institute of Medical Sciences, Patna, IND

**Keywords:** maldi-tof, sensitivity, histopathology, diagnosis, fungal infection, mucormycosis

## Abstract

Background

Mucormycosis, attributed to a group of molds known as mucormycosis, is a rare yet life-threatening fungal infection often colloquially referred to as black fungus. While its incidence notably surged during the second wave of COVID-19 infections in India, it's essential to recognize that mucormycosis was a significant concern even before the advent of the pandemic. Understanding the prevalence and characteristics of this infection in the pre-COVID era provides a crucial context for evaluating its impact and dynamics during the pandemic. Multiple diagnostic methods, such as potassium hydroxide (KOH) mount, culture, matrix-assisted laser desorption ionization-time of flight mass spectrometry (MALDI-TOF MS), and histopathological examination (HPE), are available for identifying this lethal infection. The primary objective of this study is to ascertain the sensitivity of various diagnostic methods for mucormycosis and to analyze the comparative effectiveness of microbiological versus histopathological diagnoses.

Methods

We conducted a retrospective observational study spanning six months, from May 2021 to October 2021, encompassing all mucormycosis cases meeting the inclusion criteria and diagnosed via histopathological examination (HPE) in the departments of pathology and microbiology. Microbiological tests were performed prior to the histopathological examinations. Sensitivity was assessed through statistical analysis, and the relationship between microbiological and histopathological diagnoses was evaluated using the chi-square test.

Results

Biopsy samples of 77 patients were collected, comprising 56 male and 21 female patients. Regarding age distribution, most patients fell within the 41-60 age bracket, while the smallest proportion was over 60 years old. The sensitivity and specificity of histopathological diagnosis, confirmed with periodic acid-Schiff (PAS) and Grocott-Gomori's methenamine silver (GMS) staining, both recorded a flawless 100%. KOH mount sensitivity stood at 88.3%, while fungal culture and MALDI-TOF exhibited sensitivities of 75.3%. Histopathological analysis revealed that 17% of cases displayed minimal fungal hyphae alongside necrotic tissue, whereas 58% exhibited abundant fungal hyphae accompanied by inflammatory cells. Additionally, absolute neutrophilia was observed in 55% of cases.

Conclusions

In our study, histopathology and KOH mount emerged as not only compassionate but also cost-effective diagnostic tools for identifying mucormycosis. The economic aspect of these diagnostic methods is highlighted in the results section to provide a comprehensive understanding of their cost-effectiveness. Additionally, we utilized MALDI-TOF MS as a straightforward, economically viable, and expeditious method specifically for confirming the fungal subtype in mucormycosis cases. The rationale behind choosing either MALDI-TOF MS or KOH for the diagnosis is elucidated, contributing to a clearer interpretation of our diagnostic approach. Furthermore, our findings indicate that absolute neutrophilia consistently manifests in 55% of mucormycosis cases.

## Introduction

Mucormycosis, a relatively rare yet potentially life-threatening infection when not treated promptly, is caused by a group of fungi known as mucormycetes. Commonly referred to as black fungus, mucormycosis has seen a significant surge, particularly during India's second wave of COVID-19. As of July 2021, there were at least 45,000 reported cases, prompting several states to declare it an epidemic and a notifiable disease to national health authorities [1].

Several factors have been attributed to the rise of mucormycosis in COVID-19 patients, including uncontrolled diabetes, the excessive use of corticosteroids, prolonged stays in intensive care units, and industrial-grade oxygen. Mucormycosis can manifest as angioinvasion, resulting in rapid dissemination and clinical presentation as rhino-orbit-cerebral mucormycosis (ROCM). It can manifest as pulmonary, disseminated, cutaneous, and gastrointestinal infections [2].

From a diagnostic perspective, mucormycosis remains a formidable challenge, necessitating a high degree of suspicion based on the host's immune status and clinical manifestations, which, in turn, drives further investigations for accurate diagnosis [3,4]. Microbiological tests, such as culture and potassium hydroxide (KOH) mounts, should be performed on the same tissue biopsy specimens preserved in normal saline to ensure completeness. The KOH mount, a straightforward and cost-effective method, is crucial for early diagnosis and timely treatment with specific antifungal drugs to avert fatal outcomes. Although culture is the gold standard, sporulation failure can hamper its efficacy, often yielding negative results due to non-viable organisms in necrotic tissues [3-5]. Distinguishing between live and dead organisms can be challenging since they may appear identical under microscopic examination, making culture results inconclusive. Consequently, histopathological examination (HPE) remains the sole definitive diagnostic method [3]. HPE is rapid and cost-effective for definitively diagnosing invasive fungal infections [1]. Diagnosis relies on observing non-septate, broad, wide-branched, angled ribbon-like hyphae on HPE, further confirmed through periodic acid-Schiff (PAS) staining. Therefore, the histopathological examination of mucor-like hyphae in the affected tissues is the only means to establish mucormycosis [4,5].

However, morphological identification of the hyphae cannot distinguish between different fungal species. This is crucial for selecting appropriate antifungal treatment, given that the sensitivity to antifungal agents varies among species. Matrix-assisted laser desorption ionization-time of flight mass spectrometry (MALDI-TOF MS) has emerged as a valuable alternative to molecular methods for species identification. It offers advantages such as ease of use, cost-effectiveness, speed, and high-throughput capabilities [5]. In contrast, molecular identification entails DNA extraction, polymerase chain reaction (PCR), sequencing, and time-consuming result analysis, rendering it costly [6]. No specific serological tests are available for mucormycosis diagnosis, and radiological methods lack specificity.

To effectively manage this severe complication of COVID-19, accurate identification of mucormycosis is imperative. Achieving this requires adopting a precise diagnostic approach that is rapid, cost-effective, and highly sensitive. Therefore, the objective of this study is to assess the sensitivity of various diagnostic methods for mucormycosis and to analyze the effectiveness of microbiological examinations, including KOH mounts, fungal cultures, and MALDI-TOF, by comparing them with histopathological diagnosis using special stains such as periodic acid-Schiff (PAS) and Grocott-Gomori's methenamine silver (GMS).

## Materials and methods

Study design and patient selection

We conducted a retrospective analytical study on 77 cases of mucormycosis, all of which had HPE specimens received by the Department of Pathology at our Institute between May 2021 and October 2021, spanning six months. The study was conducted at the Department of Pathology, Indira Gandhi Institute of Medical Sciences, Patna, India. It's important to note that our institution is the sole tertiary care facility in the state exclusively dedicated to treating COVID-19 complications during this time frame. All cases included in this study met the predefined inclusion and exclusion criteria.

Inclusion and exclusion criteria

Inclusion in our study necessitated the presence of mucor-like hyphae in the HPE specimens, verified through PAS staining, as determined by the pathology department. Cases with evidence of other fungal or bacterial infections were excluded from the study.

Data collection

We collected demographic data for all cases from hospital medical records and patient files. Histopathological findings were confirmed through PAS staining for all cases exhibiting mucor-like hyphae. When the pathology records lacked sufficient data, tissue slides from the histopathology department were retrieved and re-evaluated.

Specimen handling and evaluation

Specimen handling and evaluation involved a confirmation process for all cases diagnosed with mucor-like hyphae on HPE, utilizing PAS staining. In addition, we meticulously examined specimen details, KOH mounts, fungal cultures, and MALDI-TOF results obtained from the microbiological department. For KOH detection, tissue samples were subjected to a potassium hydroxide mount procedure. Fungal cultures were meticulously performed using Sabouraud's Dextrose Agar (SDA) media [7]. MALDI-TOF mass spectrometry was carried out using VITEK® MS and VITEK®2 (BioMérieux, Marcy-l'Étoile, France) instruments in the microbiological department [8]. Our assessment included an evaluation of the limited sensitivity of various diagnostic methods, and we specifically investigated the association between the absence of fungal growth on culture and tissue necrosis or infarction. Additionally, we calculated the correlation between the observed absolute neutrophilia in the complete blood count (CBC) results and the severity of the cases.

Ethics approval and consent to participate

Written informed consent was obtained from each participant after a careful explanation of the concept and purpose of the study. The participants were assured of privacy and confidentiality. The study protocol was reviewed and approved (Approval Number: /Acad., dated June 23, 2021).

Statistical analysis

We employed Epi-Info software to analyze the data obtained in this study statistically. Descriptive statistics were used to present the data, including counts (n) and percentages (%), as well as mean, standard deviation (SD), and range (min-max) values. In our analytical study, we utilized the chi-square test to correlate the percentage of association between the outcomes of fungal culture and histopathological findings, both of which involve categorical data. A p-value of less than 0.05 was considered statistically significant.

## Results

A total of 77 biopsy samples were received by the pathology department during the study's duration. Among these 77 patients, 56 (72.7%) were males, and 21 (21%) were females. Regarding age distribution, most patients (38%) fell within the 41-60 years age group, while a smaller proportion (16%) were over 60 years old. In terms of specimen distribution, the highest number of samples (60%) were obtained from crusts in the nasal cavity, while the lowest number of samples (1.3%) originated from corneal buttons (Table 1).

**Table 1:** Demographic data of mucormycosis cases

Demographic data	Frequency (n)	Percent (%)
Gender		
Male	56	72.7
Female	21	27.3
Age		
< = 40	23	29.9
41 - 60	38	49.4
> 60	16	20.8
Specimen site		
Crust from the nasal cavity	60	77.9
Sample from the maxillary sinus	14	18.2
Tissue from the cerebrum	2	2.6
Corneal button	1	1.3

The sensitivity of histopathological diagnosis, confirmed through PAS and GMS staining, was found to be 100% (Table 2). The sensitivity of the KOH mount was 88.3%. Fungal culture exhibited a sensitivity of 75.3%, and MALDI-TOF sensitivity was also 75.3% (Table 2).

**Table 2:** Showing sensitivity of different diagnostic tests for mucormycosis

	Frequency (n)	Percent Sensitivity (%)
Histopathological diagnosis after confirmation on periodic acid-Schiff (PAS) and Grocott-Gomori's methenamine silver (GMS)		
Few fungal hyphae with necrosis	17	22.1
Plenty of fungal hyphae with inflammatory cells	60	77.9
Microbiological test potassium hydroxide (KOH-Mount)		
Fungal element seen (FES)	68	88.3
No Fungal element seen (NFES)	9	11.7
Fungal culture		
No growth	19	24.7
Growth Rhizopus species	58	75.3
Matrix-assisted laser desorption ionization-time of flight mass spectrometry (MALDI-TOF MS)		
Rhizopus microsporus complex	14	24.1
Rhizopus oryzae complex	44	75.9

A chi-square test was employed to examine the association between culture outcomes and histopathological findings. A contingency table was prepared to assess whether tissue necrosis influenced culture positivity. With one degree of freedom and a significance level of 0.05, the null hypothesis was rejected if the chi-square value exceeded 3.84 (Table 3).

**Table 3:** Contingency table (observed value) for chi-square tests to find out the association between fungal culture outcomes and histopathology

	Histopathology		Total	Chi-square value	Pearson chi-square value
	Plenty of fungal hyphae with inflammatory cells	Few fungal hyphae with necrosis			
Fungal culture				3.84	66.598
No growth	2	17	19		
Rhizopus species	58	0	58		
Total	60	17	77		

The Pearson chi-square test value was 66.598, leading to rejecting the null hypothesis of independence. It was statistically significant that "no growth" on culture was associated with necrosis observed through histopathological examination. Consequently, it can be concluded that the two categorical variables, fungal culture and histopathology, are related. In our current study, we observed that most patients presented with absolute neutrophilia, characterized by neutrophil counts exceeding 7,000 per microliter, in 55% of cases (Table 4).

**Table 4:** Frequency of neutrophilia in mucormycosis

	Frequency	Percent (%)
Normal	20	26.0
Neutrophilia	55	71.4
Neutropenia	2	2.6
Total	77	100.0

**Figure 1:**
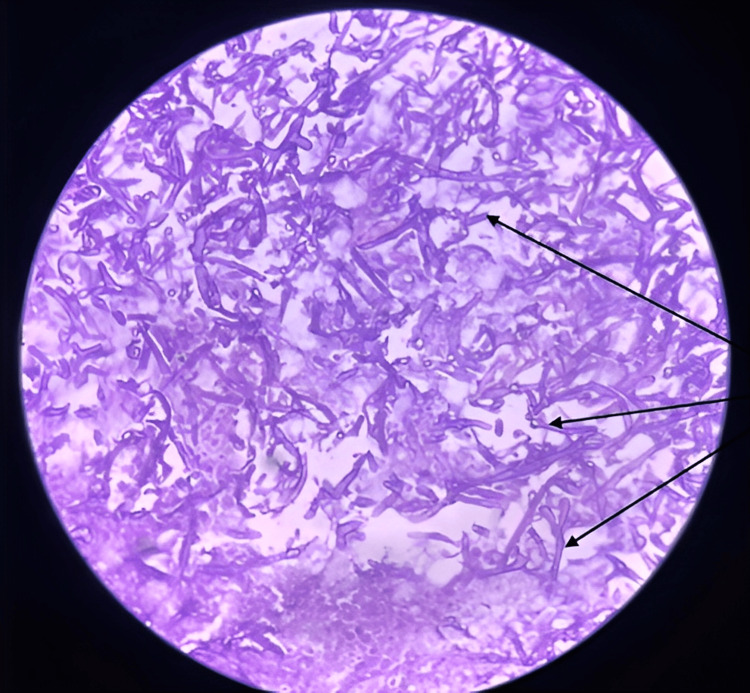
Showcases non-septate hyphae resembling mucor organisms amidst a background of extensive cell debris and inflammatory cells, magnified at 40X

Figure 1 presents an HPE slide stained with hematoxylin and eosin (H&E) dye. Under the microscope at 40X magnification, the slide vividly displays the presence of non-septate hyphae, which closely resemble mucor organisms. These hyphae are set against a backdrop of extensive cell debris and many inflammatory cells, providing a detailed view of the tissue damage caused by mucormycosis.

**Figure 2:**
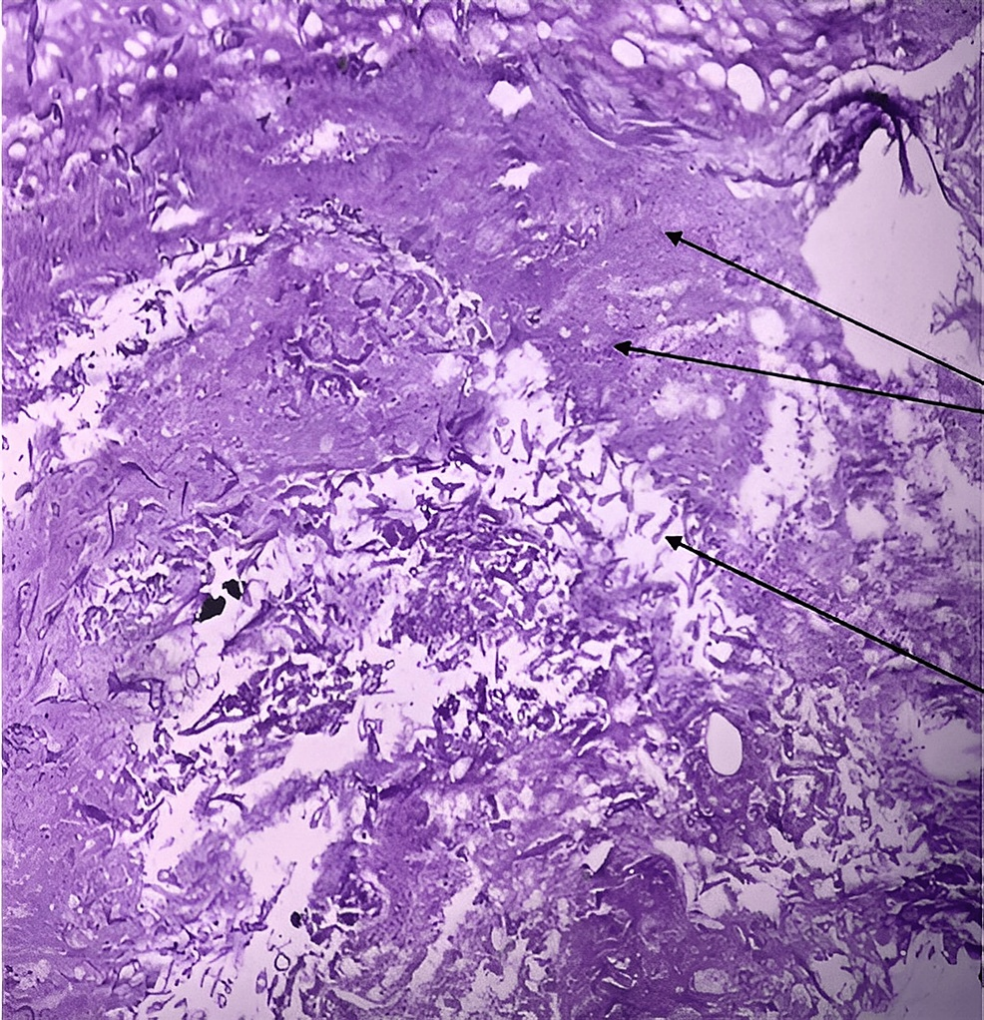
Non-septate hyphae resembling mucor organisms against a backdrop of substantial cell debris and inflammatory cells, magnified at 10X

Figure 2 was another HPE slide, but it has been stained with PAS this time. At a lower magnification of 10X, this image also reveals the presence of non-septate hyphae resembling mucor organisms. Much like in Figure 1, the background is filled with a substantial amount of cell debris and inflammatory cells, further highlighting the extent of tissue damage caused by the fungal infection.

**Figure 3:**
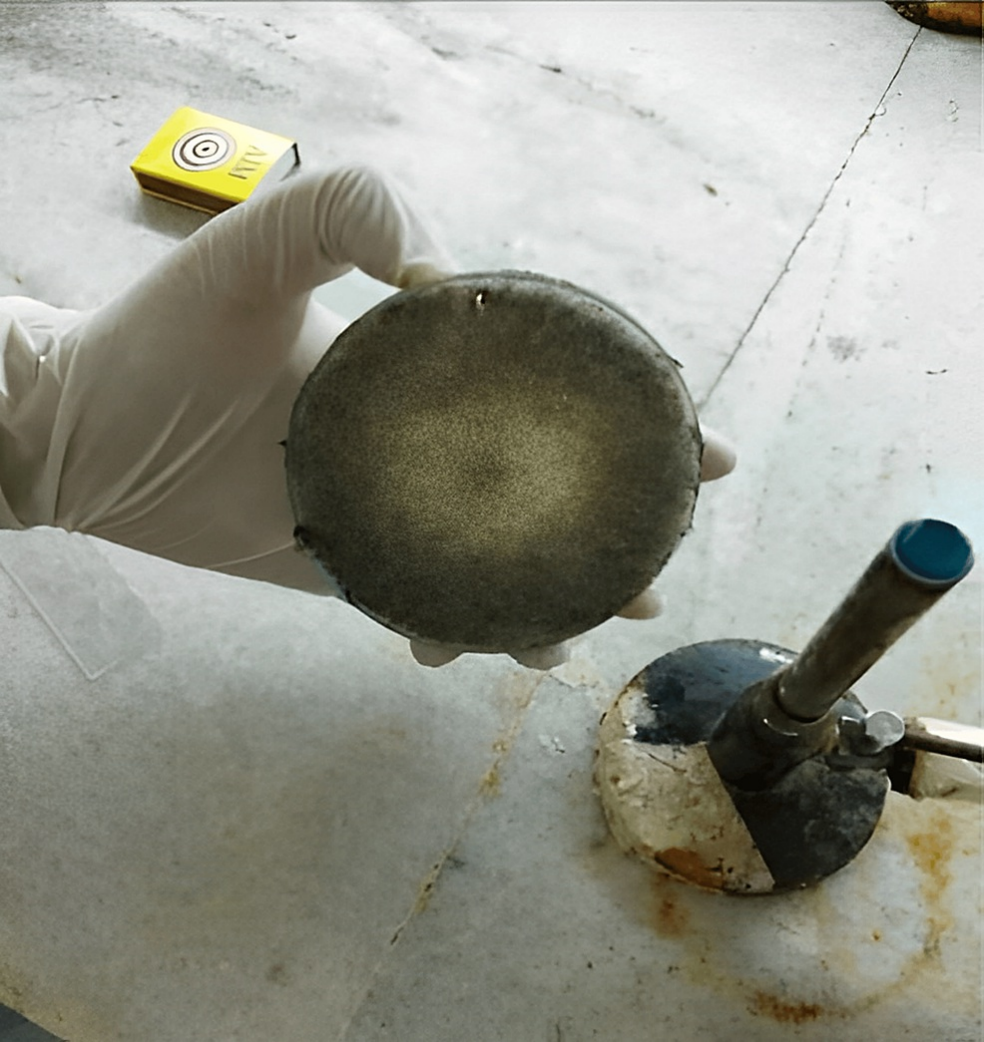
Exhibits the growth of mucormycosis on Sabouraud's Dextrose Agar (SDA)

Figure 3 presents us with a different perspective, showing the growth of mucormycosis on Sabouraud's Dextrose Agar (SDA). This image illustrates the ability of the fungus to thrive and reproduce in a laboratory culture, which is essential for diagnostic purposes and understanding its characteristics.

**Figure 4:**
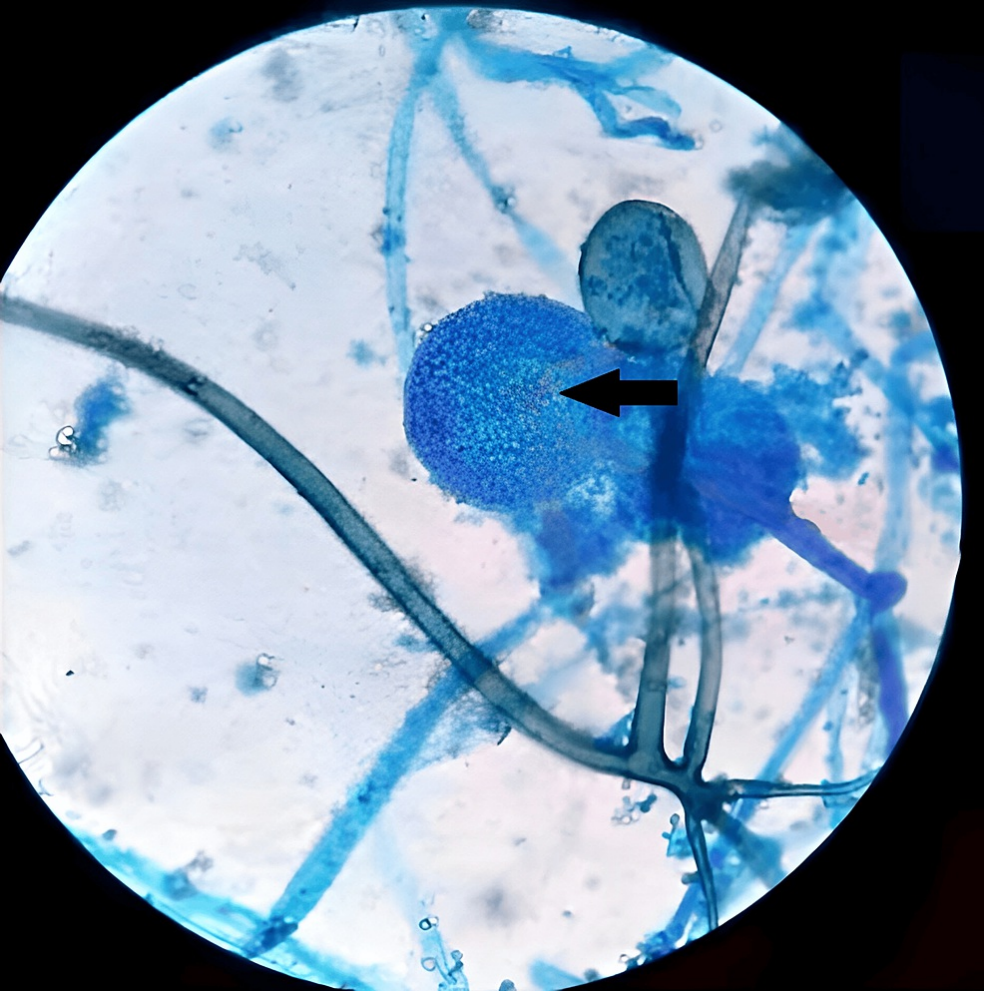
Sporangiospores in mucormycosis as observed in a potassium hydroxide (KOH) mount

Figure 4 shows a glimpse of sporangiospores within mucormycosis as observed in a potassium hydroxide (KOH) mount. These spores represent a crucial part of the fungal life cycle, aiding in the diagnosis and study of the disease. These four figures offer valuable insights into the histopathology, culture characteristics, and microscopic features of mucormycosis, enhancing our understanding of this fungal infection's pathogenesis and diagnosis.

## Discussion

Mucormycosis was first documented as a cause of human disease in 1885 [6]. Over the past two decades, there has been a notable surge in the incidence of invasive fungal infections globally. In our retrospective study, 73% of the cases were male, while 27% were female. The mean age at disease presentation fell within the 41-60 year age group (Table 1). This distribution mirrored findings from a study by Bala et al., which also revealed a higher incidence among males (72%) than females (28%), with a mean age of 40.43 years [9]. Another study conducted in North India, involving 388 proven or probable mucormycosis cases from a tertiary care center, reported a male-to-female ratio of 2.3:1 and a mean age of 45.5 years [10].

In most existing literature, rhino-cerebral mucormycosis emerged as the most common clinical presentation, a trend our study confirmed [9,10]. A review by Prakash et al. similarly identified rhino-orbital presentation as the predominant clinical manifestation associated with significant morbidity and mortality [11].

Our study indicates that histopathological diagnosis alone demonstrates a superior sensitivity rate of 100% in diagnosing mucormycosis compared to microbiological methods. The histological findings showed a robust correlation with results from the potassium hydroxide (KOH) mount (88.3%) and culture (75.3%). This finding aligns with a study by Sangoi AR et al., which reported an accuracy rate of 79% for histopathological diagnosis [12]. Therefore, our results suggest that histopathological tests, particularly PAS/GMS staining, exhibit a high accuracy rate, making them a valuable and potentially sufficient means for confirming the diagnosis of mucormycosis.

The direct KOH mount proved to be an uncomplicated, rapid, and cost-effective diagnostic test that required minimal technical expertise [13]. Our present study yielded a KOH sensitivity of 88.3%, which resonated with the report by Dass SM et al., who found a KOH mount sensitivity of 83.02% [14]. It is worth noting that morphological identification of hyphae cannot distinguish between different fungal species [15,16]. The diagnosis and treatment of mucormycosis pose considerable challenges due to the frequent lack of sporulation under standard laboratory conditions, leading to negative culture results from necrotic biopsy tissues, a trend reflected in our results (Table 3). The association observed was highly significant X2>3.84, underscoring the strong influence of necrotic tissue on positive culture findings.

The direct KOH mount proved to be an uncomplicated, rapid, and cost-effective diagnostic test that required minimal technical expertise [13]. Our present study yielded a KOH sensitivity of 88.3%, which resonated with the report by Dass SM et al., who found a KOH mount sensitivity of 83.02% [14]. It is worth noting that morphological identification of hyphae cannot distinguish between different fungal species [15,16]. MALDI-TOF MS emerged as a reliable and swift method for species identification within mucorales. Some literature has indicated that various species exhibit different antifungal susceptibility profiles [17-20]. Identifying the species level is vital for a better understanding of disease epidemiology and the future refinement of therapeutic strategies. In our study on species identification via MALDI-TOF, among the 58 culture-positive cases, the majority (44 cases) were attributed to the Rhizopus oryzae complex, followed by the Rhizopus microsporus complex (14 cases). The association observed was highly significant X2>3.84, underscoring the strong influence of necrotic tissue on positive culture findings.

While acknowledging the limitations of MALDI-TOF, such as its dependency on culture-positive samples and the time required for culture, it is essential to emphasize its unsuitability for direct tissue or fluid samples due to the necessity of combining the matrix with growing cultures for ionization. Despite the rapid detection capability of MALDI-TOF MS, the time expended on culture and its inherent low sensitivity collectively diminish its overall efficacy. Consequently, our study allows us to assert that histopathological diagnostic methods not only exhibit greater sensitivity but are also less time-consuming compared to microbiological tests. To provide a comprehensive understanding of the patients in our study, we acknowledge the need to include information on their clinical symptoms, the duration of symptoms, and the interval between the onset of symptoms and the diagnosis of mucormycosis. Additionally, the outcomes of the disease, including the survival or mortality rates, will be detailed. This comprehensive approach aims to enhance the overall clarity and clinical relevance of our study. In a related context, Diamond et al. identified neutropenic patients at an elevated risk of developing mucormycosis. However, our study (Table 4) revealed a different observation, with absolute neutrophilia observed in 55% of cases [21]. These nuanced clinical findings underscore the importance of considering various factors influencing mucormycosis development within distinct patient populations.

Limitations of the study

The limitation of our study is the relatively small sample size. Additionally, the MALDI-TOF database is restricted to a limited number of species. Expanding the database to include more clinical and environmental isolates representing different species could enhance accuracy in the future.

## Conclusions

Mucormycosis poses a grave threat as a fungal infection with an exceptionally high mortality rate, underscoring the urgency of swift diagnosis and early intervention. While the potassium hydroxide (KOH) mount is a cost-effective and invaluable tool for a rapid presumptive diagnosis, our study acknowledges the conventional view that culture is the gold standard for diagnosing fungal infections. However, it is important to note that our investigation reveals an association between histopathological findings of necrosis and the absence of fungal growth in culture, indicating potential limitations in the accuracy of culture-based diagnosis. Therefore, the combined utilization of histopathology and the KOH mount emerges as a highly sensitive and cost-effective approach to diagnosing mucormycosis. Furthermore, our study highlights a noteworthy observation: a considerable proportion of fungal infection patients demonstrated absolute neutrophilia in their CBC results. Nevertheless, recognizing the evolving landscape of diagnostic methodologies, there is a growing need for new molecular and antigenic tools to enhance early mucormycosis detection and therapeutic monitoring. In this context, MALDI-TOF stands out as a straightforward, cost-effective, and rapid method, albeit with potential considerations regarding its accuracy that merit further investigation.
